# P-32. Immunity to Measles, Mumps and Rubella (MMR) among Healthcare Workers and Health Examinees: Effect of MMR Booster Vaccination Program

**DOI:** 10.1093/ofid/ofae631.239

**Published:** 2025-01-29

**Authors:** Liang-En Hwang, Kuan-Yin Lin, Ping-Huei Tseng, Shao-Yi Cheng, Ming-Ju Hsieh, Yu-Tsung Huang, Sung-Ching Pan, Han-Mo Chiu, Yee-Chun Chen

**Affiliations:** National Taiwan University Hospital, Banciao, New Taipei, Taiwan (Republic of China); National Taiwan University Hospital, Banciao, New Taipei, Taiwan (Republic of China); National Taiwan University Hospital, Banciao, New Taipei, Taiwan (Republic of China); National Taiwan University Hospital, Banciao, New Taipei, Taiwan (Republic of China); National Taiwan University Hospital, Banciao, New Taipei, Taiwan (Republic of China); National Taiwan University Hospital, Banciao, New Taipei, Taiwan (Republic of China); National Taiwan University Hospital, Banciao, New Taipei, Taiwan (Republic of China); National Taiwan University Hospital, Banciao, New Taipei, Taiwan (Republic of China); National Taiwan University Hospital, Banciao, New Taipei, Taiwan (Republic of China)

## Abstract

**Background:**

Healthcare personnel (HCP) are considered at higher risk for measles, mumps, and rubella (MMR) infections than the general population. A national policy of MMR booster vaccination, targeting HCP born in or after 1981, was introduced in 2013, with the primary aim to boost weaned herd immunity against measles. The study aimed to evaluate the effect of MMR booster vaccination program a decade after policy implementation.

Tabel 1. Baseline characteristics and MMR serostatus among included participants

**Figure:**
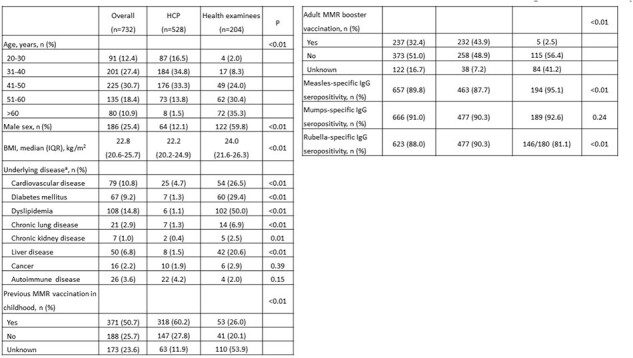

Abbreviations: BMI, body-mass index; HCP, healthcare personnel; IQR, interquartile range; MMR, measles, mumps and rubella

aCardiovascular diseases included coronary artery disease, hypertension, valvular heart disease, and arrhythmia. Chronic lung diseases included emphysema, asthma, and chronic obstructive pulmonary disease. Liver diseases primarily comprised of fatty liver disease and hepatitis B. Autoimmune diseases denoted autoimmune thyroid disease, systemic lupus erythematosus, and seronegative spondyloarthritis.

B Rubella seroprevalence data was only available in 180 of the 204 health examinees.

**Methods:**

From December 2021 to April 2023, a cross-sectional study was conducted among HCP and individuals undergoing health examinations (health examinees) at a medical center to assess the seroprevalence of MMR. The MMR booster program was started in 2013, offering a free-of-charge single-dose MMR vaccine to at-risk HCP. Baseline information and previous vaccination history were obtained. MMR-specific IgG antibodies were determined using chemiluminescence immunoassays.

**Table 2.**
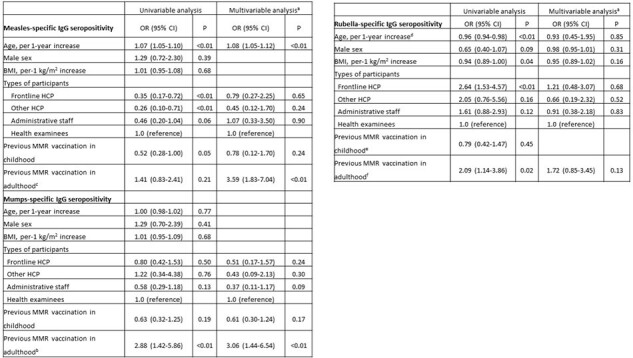
Factors associated with MMR seropositivity Abbreviations: BMI, body-mass index; Cl, confidence interval; HCP, healthcare personnel; MMR, measles, mumps and rubella; OR, odds ratio aThe ORs are the estimates of the effect of covariates on measles-specific antibody seropositivity, adjusted for age, types of participants, and previous MMR vaccination in childhood and adulthood in a logistic regression model. The ORs are the estimates of the effect of covariates on mumps-specific antibody seropositivity, adjusted for types of participants and previous MMR vaccination in adulthood in a logistic regression model. The ORs are the estimates of the effect of covariates on rubella-specific antibody seropositivity, adjusted for age, sex, BMI, types of participants, and previous MMR vaccination in adulthood in a logistic regression model. bcThose whose vaccination status was unknown were excluded from logistic regression, leaving 559 subjecs included in childhood MMR and 610 subjects in adulthood MMR. d There are 708 subjects with available rubella serostatus in total. ef There are 546 subjects with available childhood MMR vaccination history, 592 subjects with adulthood MMR immunization history with known rubella serostatus.

**Results:**

During the study period, a total of 737 participants were included, comprising 533 (72.3%) HCP and 204 (27.7%) health examinees. The median age was 43 years, with 74.6% being female. The overall seropositivity rates for measles, mumps and rubella were 89.8%, 90.4%, and 88.1%, respectively. In multivariable analysis, older age was found to significantly increase measles seroprevalence (aOR, 1.08 per 1-year increase; 95% CI 1.05-1.12). MMR booster vaccination had a significant effect on the seroprevalence of measles (aOR, 3.59; 95% CI, 1.83-7.04) and mumps (aOR, 3.25; 95% CI, 1.60-6.59), but not rubella (AOR, 1.72; 95% CI, 0.85-3.45). In the target population of booster vaccination, the uptake rate was at least 64.4%. The corresponding booster uptake rate was 17.6% for HCP born before 1981 compared to 64.8% for those born in and after 1981, and 3.9% vs 2.3% among health examinees. The seroprevalence of measles among HCP born before and after 1981 was 94.9% vs 91.9%, while among health examinees, it was 96.1% and 100%.

**Figure 1.**
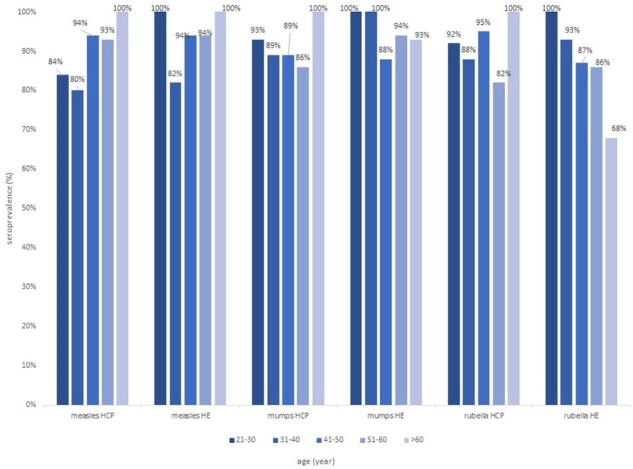
Seroprevalence of MMR among HCP and health examinees by age Abbreviations: HCP, healthcare personnel; HE, health examinee; MMR, measles, mumps and rubella

**Conclusion:**

The overall seroprevalence of MMR was high, exceeding 80% across all age groups. The introduction of MMR booster vaccination led to a rise in the seroprevalence of MMR. Our findings support the implementation of MMR booster programs among HCP and highlight the importance of enhancing the uptake rate of boosters among the targeted HCP.

**Table S1.**
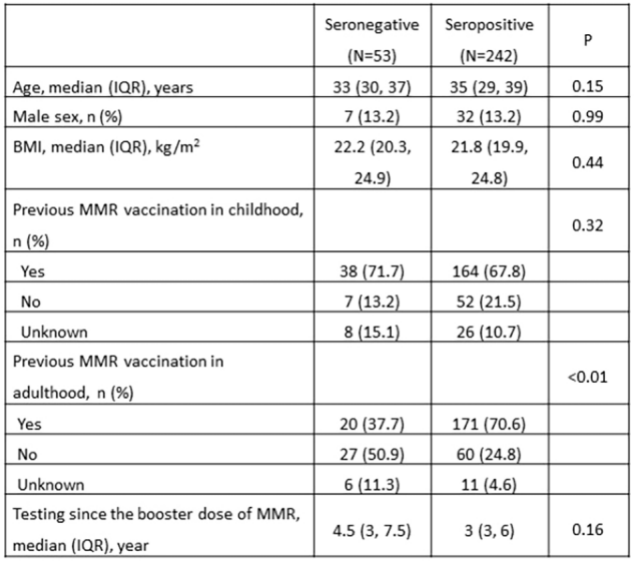
Characteristics of 295 HCP born after 1981 who tested positive or negative for measles-specific antibody

**Disclosures:**

**All Authors**: No reported disclosures

